# Design and Development of an Intelligent Skipping Rope and Service System for Pupils

**DOI:** 10.3390/healthcare9080954

**Published:** 2021-07-29

**Authors:** Yenan Dong, Kexin Wang, Shangshang Zhu, Wenjie Li, Peiyu Yang

**Affiliations:** 1 School of Design and Architecture, Zhejiang University of Technology, Hangzhou 310023, China; dongyenan@zjut.edu.cn (Y.D.); wangkx0690@gmail.com (K.W.); jawenli@zjut.edu.cn (W.L.); 2 School of Design and Technology, London College of Fashion, University of the Arts London, London WC1V 7EY, UK; ypyhz2019@hotmail.com

**Keywords:** physical activity, skip rope, children’s healthcare, mobile health care, smart mobile device, service design, user centered design

## Abstract

Regular physical activity (PA) contributes to health, growth and development in childhood and it is essential for children to achieve appropriate PA levels (PAL). However, most children around the world fail to comply with the recommended PAL requirements. Rope skipping, as a highly accessible, enjoyable, and affordable physical activity for students, has been considered a sustainable afterschool physical activity to promote physical fitness of students by educators. The booming development of smart fitness product design and the advent of exergames have brought new possibilities for physical education and rope skipping: personalized guidance, intuitive and interesting feedback and visualized exercise data analysis—there is much room for optimization. In this study, an intelligent skipping rope and its service system were studied for primary school students (aged 7–12) who started to get involved in this sport. First, user needs, product functions, and system requirement were summarized by conducting observations and user interviews. Then, a prototype of the hardware and software interface were designed based on analysis of user research. Next, a usability test of the interactive prototype was carried out and optimization was finally made based on the feedback of the usability evaluation. The final system design includes combined innovations in software and hardware with the intention to increase children’s participation in physical activity and assist them in skipping rope in the right way with proper equipment and programs.

## 1. Introduction

In 2018, the World Health Organization (WHO) launched a new global sport initiative called More Active People for a Healthier World. It is recommended that all children and adolescents should engage in at least 60 min a day of moderate-to vigorous-intensity physical activity (MVPA) [1]. However, more than 80% of students do not conform to the current guidelines for sports activities, and it is urgent to strengthen the implementation of effective policies and programs to increase activities of adolescents [2]. Rope skipping-based sports intervention can improve students’ participation in sports activities inside and outside the school and their overall physical quality [3]. Rope skipping is a whole-body exercise: the arms need to keep rotating, and the body should coordinate well with the rhythmic repetition of vertical jumping [4]. Previous studies suggested that school rope skipping has a positive impact on the psychological and physiological factors of adolescents [5,6]. In China, with the promulgation and implementation of the outline of the national fitness plan, the students’ physique standard and the national fitness plan (2016–2020) of the State Council, rope skipping has been widely promoted, and the “rope skipping plan” has been advocated by the Social Sports Guidance Center of the General Administration of Sport of the People’s Republic of China [7,8]. As an essential component of the physical fitness test in school, there is a distinct and exclusive scoring criteria for skipping rope in terms of the number of skips in one minute. For instance, for a boy in Grade 2, he could pass the test only if he skips rope more than 25 times in a minute, and the more he skips, the higher the score he gets. Such a teaching approach can teach students the basic skills of rope skipping in a relatively short time and build up distinct assessment criteria, but at the same time there remains problems and limitations: the single scoring criteria has turned rope skipping into a new test rather than an interesting activity that cultivates children’s interest in physical activity and develops self-efficacy by improving skills at their own pace.

Researchers use interactive experience design methods to stimulate children’s sports activities, such as combining sports and games to develop digital games [9]. Games can create stimulating experiences. By applying game elements such as rules, goals and rewards in non-game environments, children can be effectively encouraged to participate in sports activities [10,11,12]. Game-led learning styles and activities should be applied to stimulate students’ interest in physical activity and develop habits of physical activity [13]. With technological advances, the ability to track users’ full body movements in three dimensions, accurately measure reaction times and accelerations and capture the speed and force of the users’ movements has expanded the prospects of integrating exergames into schools and homes to promote healthy physical activity in children and decrease childhood obesity [14]. One approach that has shown some success in promoting PA in children consists of PE lessons with the Wii [15]. In another study in Latino children, an exergame named Dance Dance Revolution [DDR] was incorporated with an afterschool program. Outcomes indicated that the goal-setting strategy of exergames was effective for PA promotion among children [16]. With the increase of available smartphone applications, it is very important to document the process of designing and developing applications to enhance the credibility of mobile healthcare devices [17]. With the support of recent technological progress, the development of intelligent wearable systems (SWS) for health monitoring and fitness mobile applications is now booming [18,19]. Mobile health (mHealth) refers to the use of mobile devices (such as mobile phones and personal digital assistants) to provide health information and medical services for users [20]. High levels of engagement with mobile technology offers the access to take advantage of the potential of mobile interventions for health (mHealth) [21], including real-time data acquisition and feedback, flexibility, heart rate monitors, step counters, exercise programs and coaching guidance, and lower participant burden to resolve people’s health care needs [22,23]. Studies have confirmed the positive potential of increasing physical activity and improving health outcomes, such as management of cardiovascular disease, obesity, and type 2 diabetes under the utilization of mHealth apps and wearables [24]. At present, many studies can be found in the literature focused on the development of mobile applications in health services [17,25,26,27].

However, despite the thriving market for smart fitness products and mHealth [28], most products are oriented toward adults in China, such as Keep, FitTime and Mint health. Their main functions are to guide scientific fitness online, arrange reasonable training plans and cycles, record the user’s sports training status through big data, and then make personalized fitness program for users. In contrast, the existing intelligent fitness product designs for children and adolescents is relatively lacking. Considering the behavioral and psychological characteristics of children and adolescents, specific concerns and restrictions should be taken into account when designing smart fitness [7]. To encourage primary school students to perform daily rope skipping, most schools in China still use paper record cards or sports videos of daily attendance to supervise students to complete daily exercise. This way is simple and direct, but it is lacking in fun, which is not in line with the psychological characteristics of children at this age. Therefore, the purpose of this study is to analyze, design, and implement a new rope skipping service system design to help primary school students to participate in rope skipping activities, and motivate them to skip for fitness and fun. The general idea of the study was as follows:

1. Target user research
2. Product positioning
3. Hardware design and software design
4. Interactive prototype usability testing and optimization

The rest of the paper is organized as follows: Section 2 presents the formative research and the procedure of the field study; Section 3 presents the user research results revealed over the research process; the design of the system is described in Section 4, which is complemented with a usability test and optimization of the final prototype; Section 5 presents important findings and limitations in this study; and, finally, the conclusions and future lines are drawn in Section 6.

## 2. Materials and Methods

### 2.1. Participants

The target users of this study are primary school students aged 7–12 years old. At primary school, they are introduced to skipping rope and gradually develop skipping skills. For this age group, children are in a changing array of contexts—school, home, or with peers; they need guidance and motivation from the outside world in order to acquire the skills correctly and to persevere with this exercise. This is why it is important to consider not only the needs of the pupils themselves but also parents and schools behind them when researching user needs. A total of 19 individuals, including 13 pupils and 6 parents, participated in this study. The analysis of the sample of 13 pupils is shown in Appendix A. All participants had previous learning experience in rope skipping, with the shortest learning time being 1 month and the longest learning time being 5 years. 3 participants exercised at home and the remaining 13 participants exercised outdoors. 3 of the participants had experiences in using smart fitness products such as fitness bands. The analysis of the sample of 6 parents is shown in Appendix B. All participants had experience in teaching their children to skip rope. The frequency of accompaniment in the table represents the number of times the parent now accompanies his/her child to jump rope per week.

### 2.2. Data Collection and Analysis

Observations and semi-interviews were conducted with the participants at three neighborhoods separately in Hangzhou and Shanghai in China between October 2020 and January 2021. The observations were centered on behaviors during rope skipping, while the interviews focused on attitudes and habits towards this exercise. Qualitative data were collected including participants’ opinions and experiences, as well as quantitative data relating to daily workout habits such as duration and frequency of exercise. The detailed procedure of user research is shown in Appendix C.

(1) Observation

The purpose of the observation was to observe how rope skipping ability, exercise location (indoors or outdoors) and accompaniment (alone, with parents or with peers) affect pupils’ workout behaviors. It was mainly focused on three aspects: children’s behaviors before, during and after skipping rope; parents’ behaviors, including the way they taught their children to skip rope and their methods of recording children’s workouts; and interactions between parents and children, as well as children and their peers.

**Figure 1:**
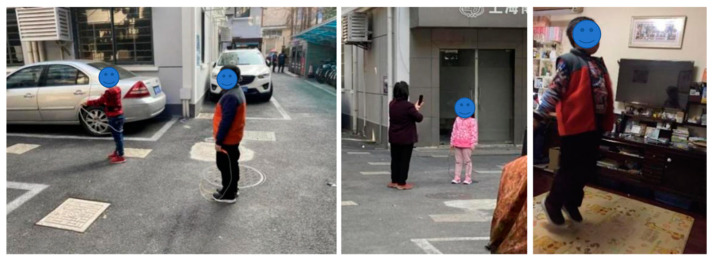
A snapshot from one of the observation sessions.

The observations were carried out in the community and in participants’ homes (Figure 1). Behaviors of pupils and their accompanists, communication and interactions between them were captured in text and photographs. The average session duration was 30 min.

(2) Semi-structured interview

The focus of the interview was not only on the rope skipping itself, but also covered aspects such as daily routine, interests and attitude towards smart fitness products. In each pupil’s interview, 15 questions of 3 aspects were asked as shown in Table 1. Pupils were asked about their daily exercise habits and goals, how they learned to skip rope and how much they knew about this exercise. They were also asked to talk about their favorite activity in PE class, their preferred games and experience in using smart fitness products such as fitness apps or fitness bracelets.

In each parent’s interview, 11 questions of 3 aspects were asked as shown in Table 2. Parents were asked to talk about the problems their children met in learning to skip rope, how school and parents motivated children to take part in sports or other activities and their attitude (expectations and worries) towards smart fitness products for children. Each interview was recorded with the permission of the participants.

## 3. Results

### 3.1. User Research Results

Based on Kairy et al. (2021) [29], an interpretive description methodology was used to carry out qualitative analysis in this study. These interviews were recorded and transcribed in a naturalized way, omitting oral expressions such as “um” and “ah” [30]. All transcripts were anonymous and recorded in Excel software. The observation and interview of data collection and preliminary analysis were carried out concurrently. Results were continuously discussed by the members of the research team [31]. Accordingly, it can be seen that the pupils need guidance and motivation in the development of skip rope skills. At school, they learn from their teachers. After school, they learn and make progress through the interaction between family and friends. Specifically, the following conclusions can be drawn:

From pupils’ aspect:

(1) Most teaching stops at the traditional two-feet basic jump

The traditional two-feet basic jump, which is to jump on two feet consistently, is the most basic and easiest way to skip rope. For most pupils, according to our interview, it is the first and only skill they learned. One mother mentioned in the interview that once her child had learned the basics of skipping rope, the exercise goal turned to increasing speed, as the only factor determining performance in school tests is the number of skips in one minute. This result is also due to the different teaching features of physical educators. In the interview, a third-grade boy said that his PE teacher had once showed them the skill of cross jump and he thought it was interesting. However, a second grader from another school said that the way he practiced skipping rope in PE class was usually a one-minute timed jump.

(2) Physical health awareness is lacking

In the observation, only 3 of 13 participants started skipping rope with a warm up and stretching exercises. The children said that they would do warm-up exercises with the teacher in PE class at school, but they didn’t know that it was essential to do warm-up exercises before skipping rope.

(3) Gamification brings fun

Students often use the word fun to predict or evaluate the worth of activities in which they engage. To find out the criteria of “fun” among pupils, interviews were focused on the attractive elements in pupils’ daily life and “game” turned out to be the “magic word” to motivate students. Teachers are now using gamification apps to assist them with teaching. The interview discovered that English teachers in primary school are now using game-based apps to help students correct pronunciation and complete listening exercises. The combination of learning tasks and games have proved to be attractive to pupils, as a parent said that her child always chose to finish the homework assigned on app first after school.

(4) Social support brings motivation

In physical activity, children may receive support from their parents, teachers and peers. Their support exerts a positive effect on children and motivates them to continue to participate in the physical activity. Support can take the form of verbal praise; a second-grade boy recalled an impressive memory that his PE teacher once praised him in front of the whole class for the progress he had made in the skipping rope test. In addition, it was observed that many parents would accompany their children to practice skipping rope in the community open area. As the child kept skipping rope, the parents would constantly give verbal encouragement to the child and would also motivate the child to keep skipping by telling them the number of skips they had completed. This was especially true for children who have not yet mastered the skill. They kept tripping over the rope, which would make them frustrated. At this time, affirmation and encouragement from parents would motivate them to persevere.

Support also includes material rewards. One father said that his first-grade son was reluctant until he promised to buy him an airplane chess if he could keep practicing skipping rope for a week. School teachers have also set up reward rules to encourage pupils to participate actively in class. A third grader said that if he performed well in class, his teacher would award him with a sticker, and he was working hard to collect 10 stickers so as to exchange them for a gift from his teacher.

From the parents’ aspect:

(1) Parents need teaching guides

In the interview, parents mentioned that they would teach their children how to skip rope in advance to get prepared for primary school study, and it was always hard for children to get the rhythm of skips at the beginning. Some parents denoted that, at this time, they would search the internet to look for some teaching videos which introduced several pre-exercises to help children grasp the rhythm. Some parents even go so far as to send their children to skipping rope training class. News reported that skip rope classes were quite popular, and some parents spent over CNY 10,000 a year for children to learn to skip rope. Skipping rope is taught not only at school, but also at home, and parents need teaching guides to better teach their children to skip rope.

(2) Recording is necessary

It was observed that parents took photos or videos while their children were skipping rope, which according to the parents, “is a school requirement to check the completion of daily exercise”. Other ways of recording included recording the number of rope skips on a paper card. In the parents’ opinion, this was also one of the homework assignments. One parent also said that the daily record gave her an idea of how her child progressed in skipping rope.

(3) Parents’ worry about smart products

When it comes to children’s use of smart products, such as mobile phones or iPads, most parents said that they strictly controlled their children’s screen time, especially on school days. As for the use of exergames, parents expressed that they would prefer their children to get outside and play sports than to work out in front of the screen. Other parents were concerned that too many game features might lead to addiction.

### 3.2. Persona

Human-centered design is a design paradigm whose focus of questions, insights and activities is on the people the product, system or service intended for [32]. Persona, the “hypothetical archetype of real users” [33], is a modelling tool to this paradigm to capture specific user needs and generate more creative and human-centered solutions [34].

**Figure 2:**
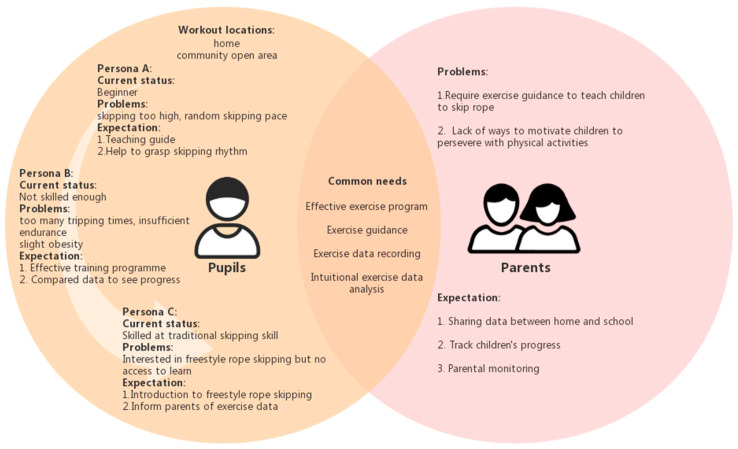
Personas of pupils and parents.

In order to clarify the needs of pupils and their parents, three pupil personas and one parent persona were established according to the user research (Figure 2). Problems and expectations of pupils were grouped into three personas based on their current skipping rope abilities and exercise goals. The persona of parents was created according to the typical problems and parental expectations reflected in the user research. Lastly, the common needs of pupils and parents are summarized.

### 3.3. Product Positioning

To ensure the accuracy and accessibility of exercise data acquisition and the integrity of system functionality, the system uses a combination of software and hardware of an intelligent skipping rope. Considering the different requirements of children and parents during rope skipping, the software is equipped with two terminals: the mobile phone terminal is for the parent and the smartwatch terminal is for children. The functional layout of the software on both terminals is slightly different depending on their different demands. The final intelligent skipping rope service system of this study includes the following functional innovations:

(1) The intelligent skip rope service system is composed of hardware products and software products. The hardware products are portable and easy to use. The interface of the software is intuitive and simple.
(2) The hardware products of the intelligent skipping rope service system consists of a smartwatch and an intelligent skipping rope. They support multichannel data acquisition of exercise data and health condition.
(3) The hardware products of the intelligent skipping rope service system also bring about interactions between terminals and between users, which improves the user experience and increase the frequency of use.
(4) The software of the intelligent skipping rope service system could generate personalized exercise plans, provide skill guidance, integrate and analyze users’ daily exercise data, and provide multifaceted feedback to encourage users to skip rope for fitness and fun.

**Figure 3:**
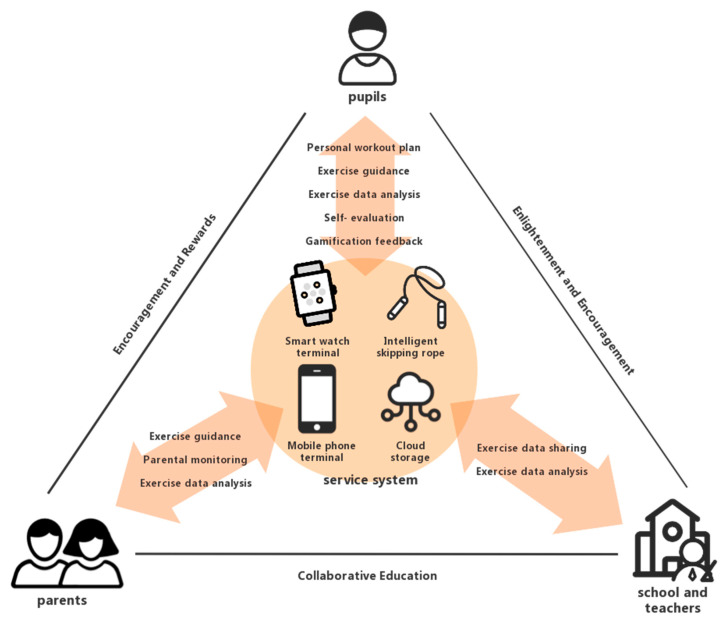
Service framework of the intelligent skipping rope system.

Combined with the existing basic research of smart fitness products and the target user research, user requirements were summarized and translated into five core functions (Figure 3).

Exercise data acquisition: In the process of rope skipping, the data to be collected include number of skips, tripping times, heart rate and duration of exercise. Tripping times could reflect whether the skipping posture is correct and whether the coordination between hand and feet has a good beat. Additionally, heartbeat offers a more objective look at exercise intensity.

Exercise data analysis: Based on the acquired exercise data, processing and analysis of the data can offer users a clearer picture of their exercise. This is especially for the pupils, for whom highly-visualized data analysis could intuitively reflect their progress. They could see how many skips they have accomplished, how fast they have become and be more motivated. Additionally, data analysis could help users realize their weaknesses and make adjustments to their plan accordingly. For instance, if a user’s tripping times remains at a high level, it means he or she might not yet grasp the rhythm and need more basic pre-exercise.

Exercise data sharing: As an after-school physical activity, the sharing of pupils’ exercise data between home and school can help schools better supervise pupils’ daily physical activity and rope skipping ability. Adjustment to teaching programs can be made based on these data. Compared with the current recording methods, software-based data flow is more effective and convenient.

Exercise guidance: Exercise guidance includes not only step-by-step instructions, but also personalized, long-term advice for improving motor skills. In schools, rope skipping is taught in classes and the content is determined by the average level of skipping, leaving the needs of individuals who are behind or ahead of the collective unmet. The guidance can be in the form of text, video, animation and audio tracks, which is a useful way to set skipping pace used by competitive speed jumpers as a training technique.

Interesting and effective incentives: Factors that affect physical activity levels (PALs) in children include psychological aspects such as self-efficacy and motivation, and sociological aspects including support from school, parents and friends [35]. Study found that children became unwilling to participate in PA when there was little enjoyment or “fun”, and by comparison, they tended to continue to participate when a high level of enjoyment was experienced during PA [35]. Csikszentmihalyi’s Flow Model (1975) described fun in a simple way, as “the balance between skill and challenge”. To make physical activity “fun” and attractive is to emphasize intrinsic aspects such as personal improvement, skill development, optimal challenge, personal goals, opportunities to get involved and constructive feedback rather than extrinsic aspects such as winning [36].

## 4. Hardware and Software Product Design

The hardware and software parts of the system were designed based on the above user demand analysis and functional positioning.

### 4.1. Hardware Product Design

With the aim to implement portability and comfortability to encourage children’s use, as well as to implement the functional connection within the whole system, the appearance and circuit implementation of the intelligent skipping rope was designed as follows.

#### 4.1.1. Hardware Appearance Design

**Figure 4:**
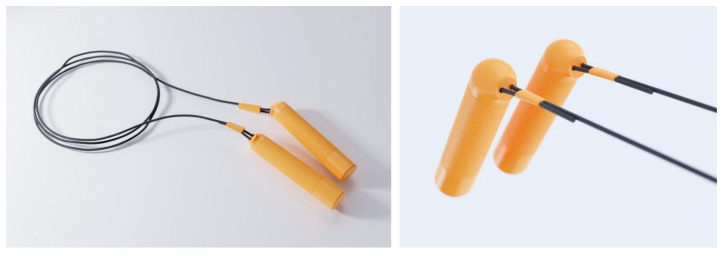
Hardware appearance design.

**Figure 5:**
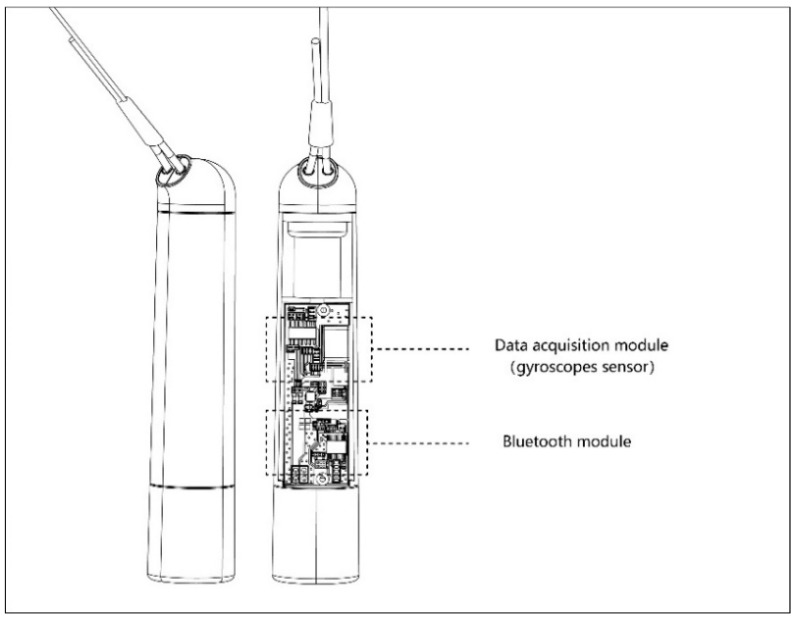
Internal structure of the intelligent skipping rope.

The design of the hardware’s appearance is shown in Figure 4 and the internal structure of the intelligent skipping rope is shown in Figure 5.

Sustainable use: The handles of the intelligent skipping rope were designed ergonomically with sufficient length so as to fit well in the hand during the child’s growth. The length of the skipping rope could also be easily adjusted to suit the child’s changing height.

Color: The orange color of the intelligent skipping rope, according to colour psychology, would evoke feelings of excitement, enthusiasm and energy [37].

Material: The soft rubber surface of the handle enhances grip comfort and absorbs perspiration well.

#### 4.1.2. Modular Implementation

**Figure 6:**
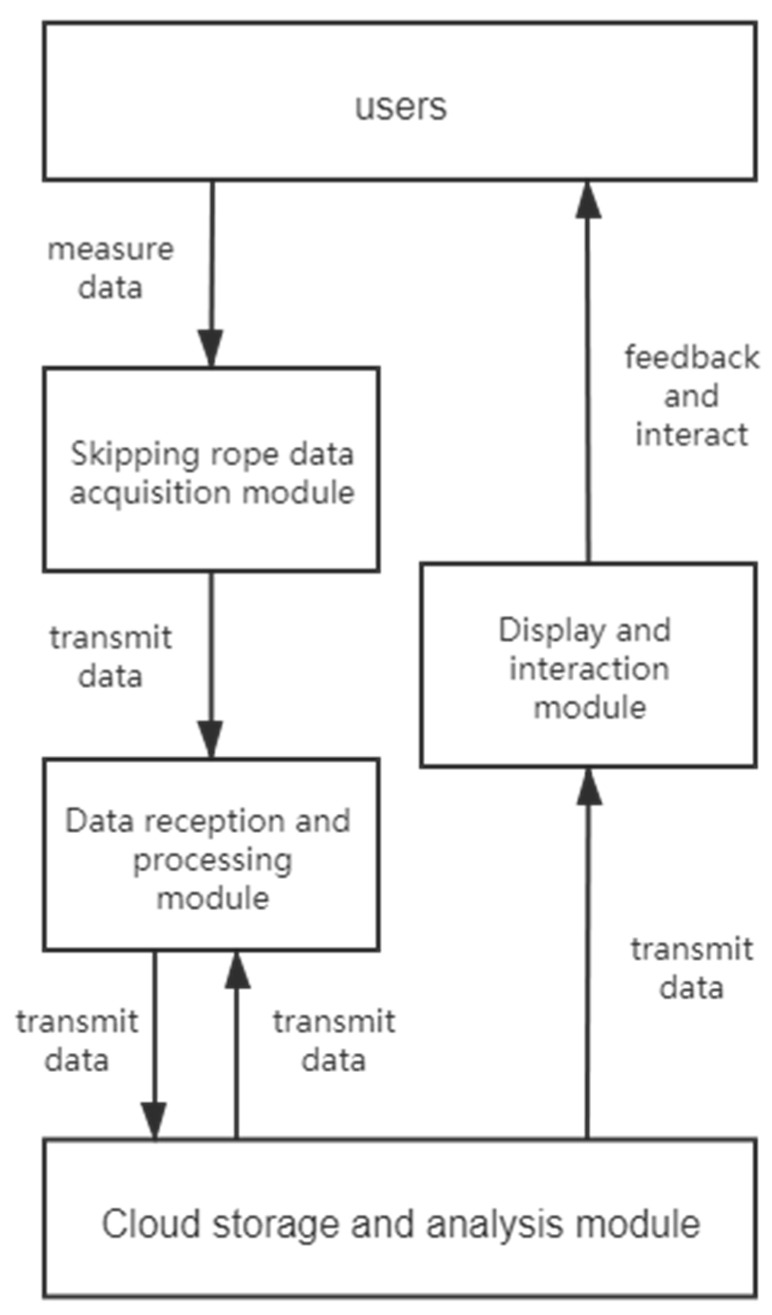
Acquisition-analysis-feedback framework.

The overall acquisition-analysis-feedback framework of the intelligent skipping rope service system is as follows (Figure 6):

(1) Skipping rope data acquisition module

The basic need of data acquisition is supported by the gyroscope sensor on the intelligent skipping rope and the heart rate sensor on the smartwatch terminal. The data collected on the intelligent skipping rope are transmitted to the data smartwatch terminal through Bluetooth for further processing.

(2) Data reception and processing module

The system uses the smartwatch terminal as the data reception- and further processing-processor to ensure the feasibility of real-time data viewing. The data reception and processing module receives the real-time data collected by the skipping rope data acquisition module and calculates and integrates simultaneously the required values of number of skips, tripping times, heart rate and duration of exercise. The data are uploaded to the server and stored in the cloud when the measurement is complete.

(3) Cloud storage and analysis module

To assure the consistency of the data on different terminals and to realize the sharing of the exercise data between family and school, each measurement of the user is stored in the cloud. The server side implements the function of data storage and analysis. In addition to measurement data tables, the server side is also responsible for the maintenance of user tables. The measurement data tables record users’ measurement results. The user tables record the basic information of each user, such as their gender, height, weight, current skipping rope ability and workout goals, which serve as a reference for plan formation.

(4) Display and interaction module

**Figure 7:**
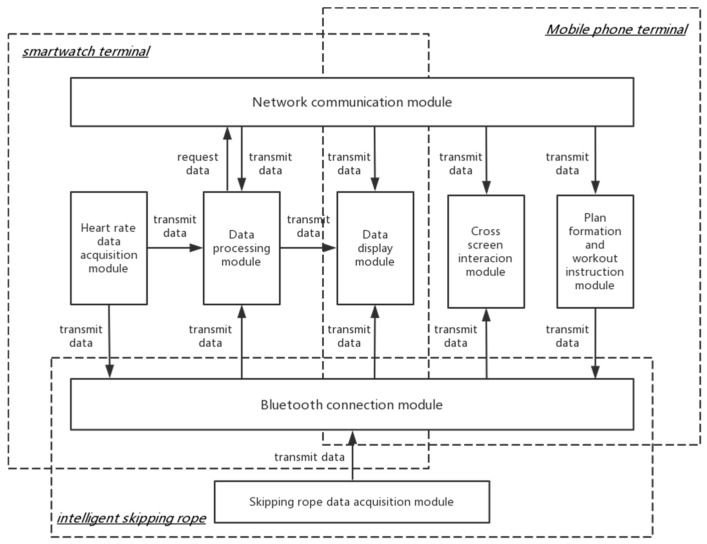
Multi-terminal skipping rope service system framework.

During a workout, the exercise data, such as number of skips and time duration, were displayed in real time on the screen of the smartwatch terminal. In the mobile phone terminal, users could get access to more detailed exercise analysis and historical measurement. Considering the interactive needs between parents and children, as well as teachers and students, the system applies web services for communications. In addition, cross-screen interactions are implemented under the appliance of the web service and the sensor on the smartwatch which could recognize the user’s hand motion. The overall multi-terminal skipping rope service system framework is shown in Figure 7.

### 4.2. Software System Interface Design

**Figure 8:**
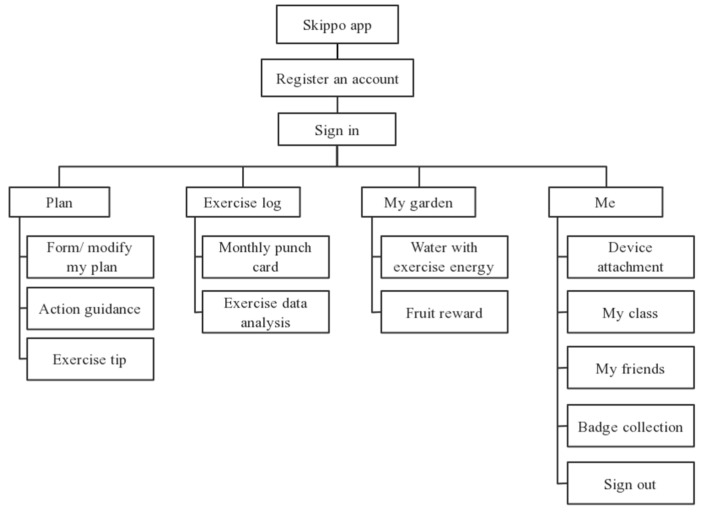
Software function flow chart.

Based on the product positioning, the functional flow chart of the software system is shown in Figure 8.

#### 4.2.1. First Round of the Interface Design

(1) Functional implementation

**Figure 9:**
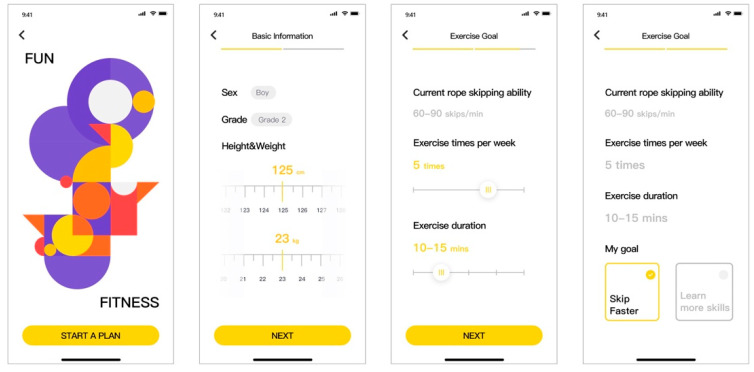
Plan formation interface.

Plan formation—First-time users enter the Plan interface on the mobile phone terminal. The interface then jumps to a plan formation page, where the users are required to enter basic information such as gender, grade, height and weight, as well as information about their current rope skipping ability level, exercise frequency and duration, and exercise goals. Skipping rope standards vary for children of different ages and the system will generate a personalized plan for the user based on the user’s input and exercise goals (skip faster or learn more skipping skills). For sustainable use, the plan formed can be modified at any time as the user’s skipping level changes (as shown in Figure 9).

**Figure 10:**
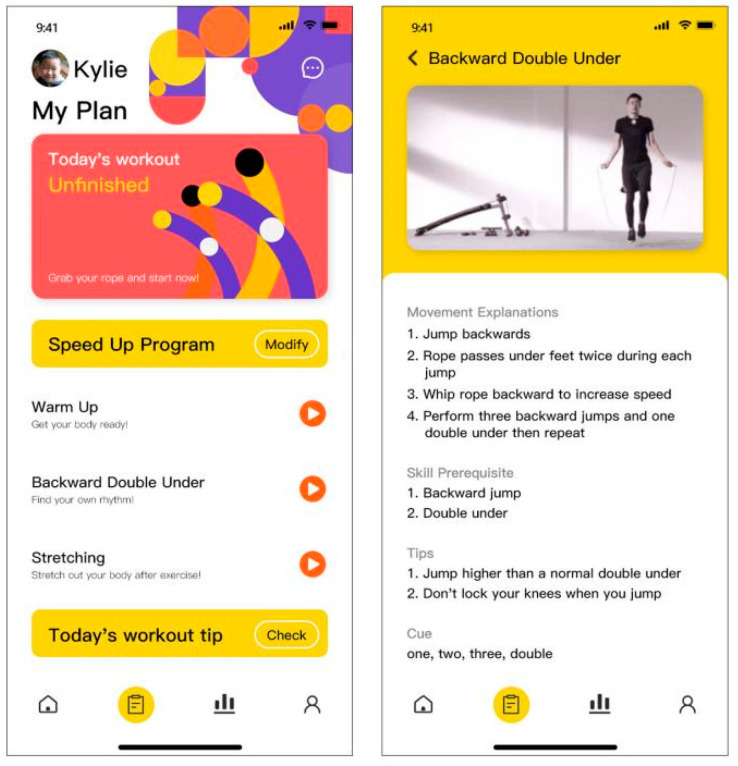
Exercise guidance.

**Figure 11:**
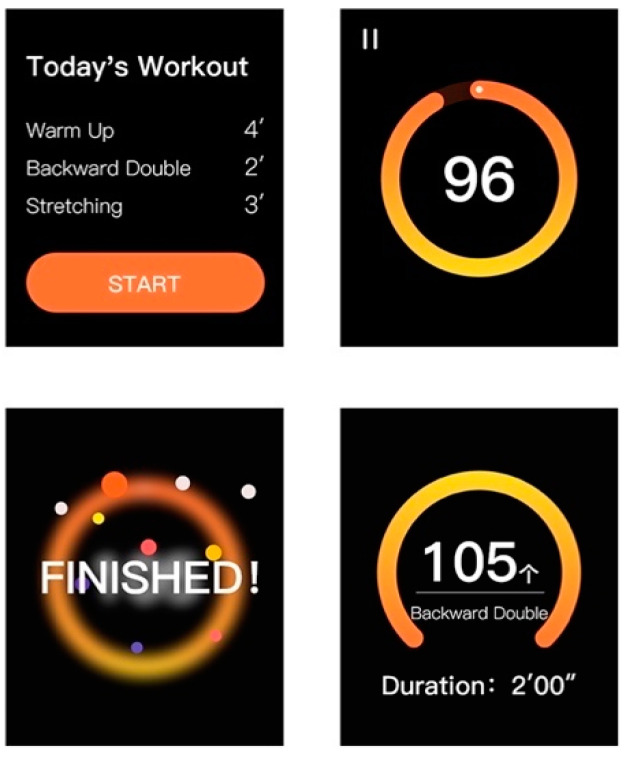
Exercise interface on smartwatch terminal.

Workout—During the workout, the interface on the mobile phone terminal is mainly for parental use and includes video demonstrations and textual posture instructions to guide their children (Figure 10). The children’s operation during workout is on the smartwatch terminal, where the user can click the button to start. The interface visually shows the real-time number of skips and time left through the time circle (Figure 11).

**Figure 12:**
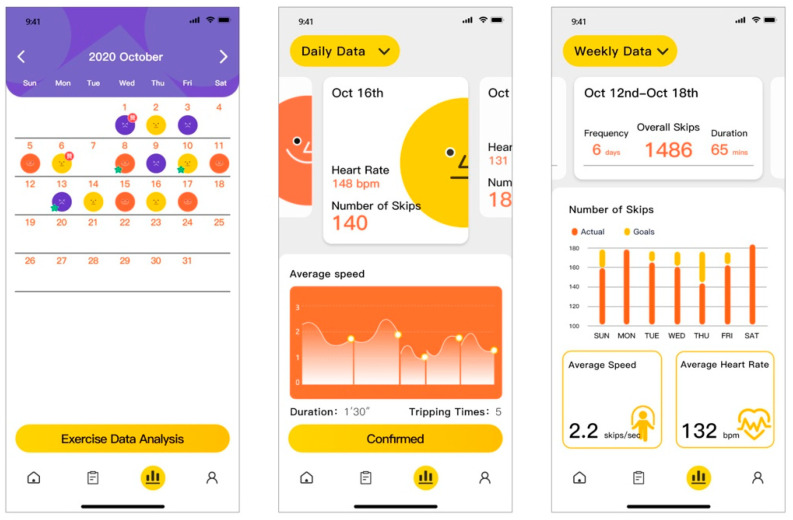
Exercise data analysis.

Exercise data feedback—After completing the workout, the interface on the smar watch terminal displays the number of skips and workout duration (Figure 12). More detailed exercise data analysis is available on the mobile phone terminal, including the number of skips, tripping times, average heart rate and speed graphs. Users can click the toggle button in the upper left corner to switch from daily data analysis to weekly analysis to see the total amount of workout and data changes over the week. By swiping left and right, the user can view historical data analysis.

(2) Incentive feedback system

Self-evaluation—After the workout, users select an emotion stamp on the smartwatch terminal to evaluate their feeling regarding the exercise. The stamp they choose is recorded in the exercise log interface on the mobile phone terminal.

**Figure 13:**
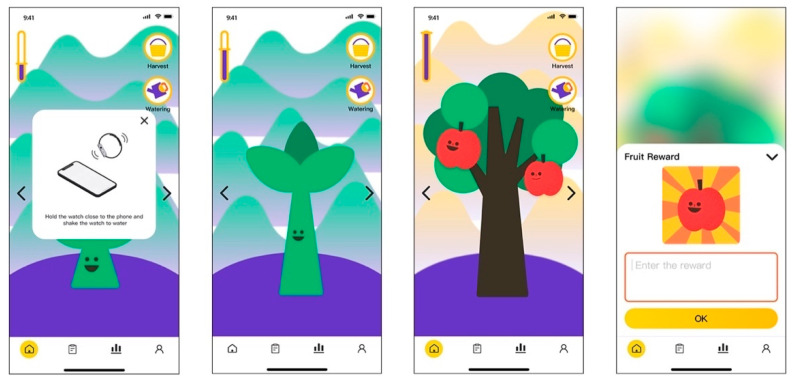
Gamification feedback: My Garden.

Gamification feedback—A tree-planting game is introduced to visualize children’s efforts and motivate them to persevere in exercising: after exercising, the watch will display the “exercise energy” accumulated through workout (Figure 13). In the interface of My Garden on the mobile phone terminal, user can consume “exercise energy” to “water” the plant by the action of shaking the smartwatch. The virtual sapling grows and eventually bears fruits. Each fruit can be exchanged for a reward from the parents, who can enter the content of the reward in the corresponding input box. Once a tree has matured, the user can unlock the next planting scenario by swiping left and right.

Parental feedback—When the user has finished the workout, a notification will appear on the mobile phone to notify parents of children’s completion. After the parent has checked their child’s exercise data and clicked the confirm button in the exercise log interface, the smartwatch receives a star sticker to remind the child that parents have given “me” a “thumb-up”.

School feedback—In order to implement home-school exercise data sharing, the user could enter the personal homepage interface, click the “My Class” button, enter the class account and join in the class group. After checking the data, the teacher could choose to give certain student a “thumb-up” as encouragement. The smartwatch would receive a red flower sticker to remind the child as a result.

Friend interaction—There is a friend interface on both the mobile phone terminal and smartwatch terminal where users could view their friends’ workout for the day and choose to give them a “thumb-up”. If the user receives a “thumb-up” from a friend, a reminder would appear on the smartwatch.

To add a new friend, the user needs to enter the friend interface, click the “add a friend” button and then shake the skip rope. Two skip ropes could recognize each other within a specific distance and, accordingly, the account information will appear on the screen of the smartwatch. Users click “confirm” to add each other as friends.

#### 4.2.2. Software Interface Usability Testing and Results

After the low-fidelity interactive prototype was finished, a usability test was conducted for the software system. In order to improve the usability of the applications, the following standards should be followed: the procedures and operations of applications must be efficient, easy to remember, easy to learn, subjectively make users feel happy, and must produce minimal errors. Seven participants, including three adults and four primary school students took part in the test. Before the test, users were introduced to the basic usage of the application. During the test, the users were required to complete 8 tasks through low-fidelity interactive prototypes. Their actions, words and expressions were recorded. Problems in the interaction, design and functional issues of the software were identified in terms of the following 8 metrics: successful task completion, critical errors, non-critical errors, error-free rate, time on task, subjective measures and likes, dislikes and recommendations [38]. The specific task schedule for usability testing is shown in Appendix D.

Based on the results of usability testing, the software interface design was optimized. The problems that needed to be optimized were the following:

- Task 1 and Task 3. The icon of My Garden was misleading: users mistook it as the Homepage. Additionally, the order of the tab arrangement did not correspond to the order of use, since the users assumed the first tab on the left was the interface to start with.
- Task 2. In the process of forming a plan, users couldn’t make changes to the content previously filled in.
- Task 4. Users expressed a need to record additional workouts after completing the daily scheduled exercise.
- Task 5. By simply displaying the heart rate value, users couldn’t determine for themselves whether their heart rate was within the normal range.
- Task 7. Users couldn’t quickly understand what the My Garden interface was for. They neither knew how to obtain the water to care for the plant nor what would happen if they kept watering the plant.

#### 4.2.3. Optimized Interface

**Figure 14:**
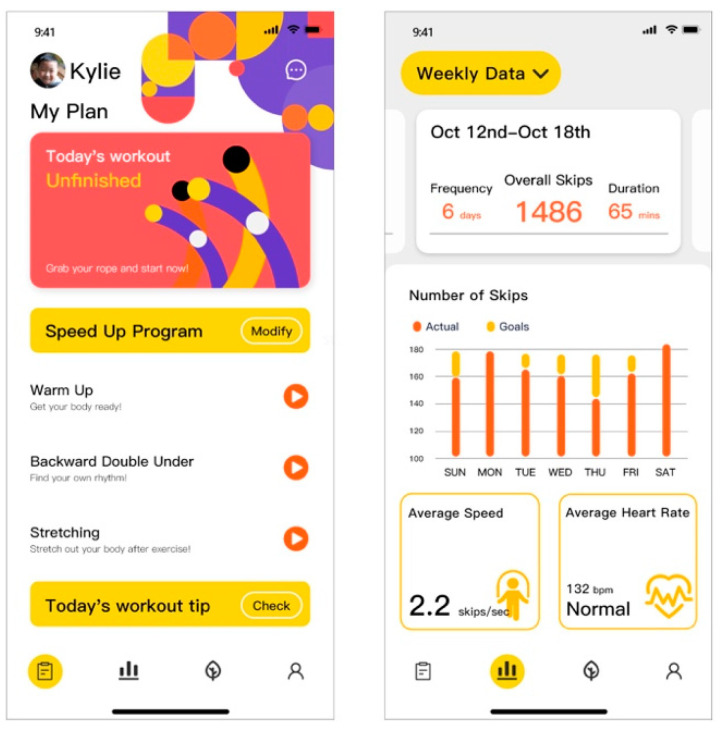
Optimized main interface and exercise log interface.

Based on the feedback from the user testing, the optimized interface design is as follows:

Main interface: According to the analysis result of Task 1 and Task 2, the order of the tabs was adjusted in accordance with the sequence of exercise data analysis—My Garden (gamification feedback)—settings. The icon of My Garden was also substituted to improve identification accuracy (Figure 14).

In the interface of plan formation, a back button was added in the upper left corner for users to jump back to revise the previous options (from the feedback of Task 2). Based on the analysis of Task 5, evaluation of heart rate was added to help users better control their exercise intensity. In addition, to offer users an intuitive overview of the My Garden interface, a description was added when users enter this interface for the first time. The description explained the gamification feedback system and the meaning of each icon in the interface (from the feedback of Task 7).

**Figure 15:**
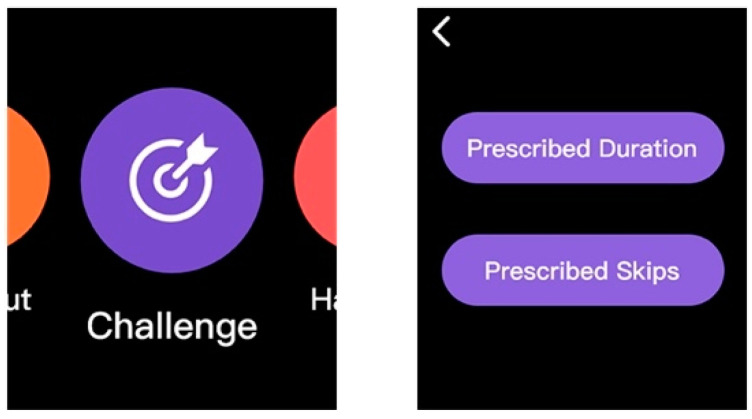
Optimized smartwatch interface.

Additional features: Based on user feedback of Task 4, a new feature called Challenge, which offers an additional workout as an alternative aside from the plan was added in the smartwatch terminal interface (Figure 15). Users can specify the time duration or the number of skips to begin with. Data analysis for additional workouts can also be accessed in the data analysis interface.

## 5. Discussion

### 5.1. User Requirements of General Importance

The intelligent skipping rope service system provides exercise guidance in the form of a personal workout plan for students with different skipping abilities. For example, students who are just starting to learn to skip rope need effective pre-exercises and detailed instructions to help them master the correct skipping posture and grasp the rhythm for skipping. On the other hand, students who want to improve their skipping speed need a scientific and effective training program to reach this end. As for students who are interested in other ways of skipping, they need expanded introduction to other freestyle skipping skills. In addition, exercise guidance enhances students’ physical health awareness, guiding them to form the habit of warming up and stretching.

To bring “fun” to the physical activity of rope skipping and to motivate children to develop physical activity into a life-long hobby, this study established a multifaceted incentive system which includes self-evaluation, support from parents, peers and school. Additionally, considering space constraints, the fact that most children skip rope outdoors and the concerns of parents about exergames, gamification features are applied in post-exercise feedback rather than throughout the entire exercise process.

### 5.2. Implications for Design Research

In the design process, designers should always take into consideration the different needs of children and parents. Given the different user scenarios, the service system designed in this research requires a combined use of a smartwatch terminal and a mobile phone terminal, the former of which orients towards pupils, while the latter is mainly for parental use. To implement the modular function and improve maintenance, the service system uses web services to realize communication between the mobile terminals and the server side.

While the intelligent skipping rope acquires exercise data, the software part of the intelligent skipping rope system supports daily exercise plan guidance and an exercise data log of the users. At the same time, it provides gamification feedback to visualize the amount of exercise to motivate users to keep exercising. In addition, it has a data sharing function which could connect family and school together; the feedback from parents, teachers and peers would serve as a source of motivation.

### 5.3. Study Limitations and Future Directions 

Several aspects limit the generality of the current research results, which can provide reference for future research. Firstly, the number of users participating in this study was limited, and more participants will improve the effectiveness of the study. Secondly, the results of this study conducted in China may be limited to a Chinese cultural background, and future studies should consider people from different cultural backgrounds. Furthermore, although qualitative approaches gave useful insights in the first phase of designing and developing the intelligent skipping rope and service system, the usability test result of the prototype is imprecise. Future studies should continue to perfect the function of the system, improve the user experience and explore the use of the system in a real-life setting.

## 6. Conclusions

Primary school students are in a critical period of physical growth. Rope skipping, as a whole-body coordinated activity, can provide students with cardiovascular fitness, strength, and conditioning. With the rapid raise of the obesity rate in school-aged children and the proposal of health-enhancing physical activity (HEPA), rope skipping is the perfect fitness activity to promote health and fitness in students. The booming development of smart fitness product design and the advent of exergames have brought new possibilities for physical education regarding rope skipping.

This research took an intelligent skipping rope service system for pupils as the research object and aimed to design a new intelligent skipping rope and a complete service system. This research summarized users’ needs through observation and semi-structured interviews, established personas, and proposed a combination of software and hardware based on production positioning. The interactive prototype was tested through usability evaluation, and optimization was then carried out according to the feedback.

The final intelligent skipping rope service system design, in the combination of hardware and software products, implemented the acquisition, analysis and sharing of the exercise data. In addition, the features of personalized plan formation with exercise guidance and the incentive system which included self-evaluation, support from parents, peers and school as well as gamification feedback, made improvements in educating pupils with the knowledge, skills, and confidence to enjoy healthy physical activity. In order to determine the effect of the intelligent skipping rope service system on pupils’ PA levels, self-efficacy, enjoyment, and psychological needs satisfaction, more experiments based on larger sample groups should be conducted as future work.

## Figures and Tables

**Table 1:** List of the questions used in interviews with the pupils.

Indicator of the Interview	Question of the Interview
Skipping rope	When did you learn to jump rope and who taught you? How often and how long do you skip rope per week? What kind of skipping rope do you use? How do you feel about it? How is skipping rope taught at school? Do you know other skipping skills in addition to the traditional two-feet basic jump? Are you interested? What is your goal in skipping rope?
Daily life (physical activity and sedentary behaviours)	What is your favourite activity in PE? What other physical activities do you usually do? Do you play sports with your parents? Are there any incentives at home or at school? How much time do you spend in writing homework every day? Do you have any smart products in your home? How often do you use it? What do you do with it? Have you ever used learning games? What do you think about it?
Experience with smart fitness products	Have you ever used any smart fitness products? Do you know exergames? Have you ever tried one?

**Table 2:** List of the questions used in interviews with the parents.

Indicator of the Interview	Question of the Interview
Skipping rope	Have you ever taught your child skipping rope? Is there a school assignment for afterschool skipping rope practice? How is the completion checked? What is your attitude towards daily skipping rope practice? What about your child’s attitude? Do you go skipping with your child? Do you keep an eye on the number of rope jumps your child makes?
Daily life (physical activity and sedentary behaviours)	Do you pay attention to your child’s daily physical activity? Would you take incentives to motivate your child? How long does your child spend on screen time every day? What is your opinion about learning games?
Attitudes towards smart fitness products	Have you ever used any smart fitness products with your child? Do you know exergames? What do you think about it?

## Appendix A

**Table A1:** Pupil characteristics.

Participant Number	Age	Gender	Exercise Location	Frequency of Exercise (Days/Week)	Practice Duration (Years)	Types of Skipping Rope	Use of Smart Fitness Product
Pu1	9	Female	Community open area	5	3	Traditional	No
Pu2	11	Female	Community open area	7	5	Traditional	No
Pu3	8	Male	Home	3–5	2	Traditional	No
Pu4	7	Male	Community open area	1–3	1 months	Traditional	No
Pu5	9	Male	Community open area	5	3	Traditional	No
Pu6	7	Female	Community open area	5	1	Traditional	No
Pu7	10	Male	Community open area	1–3	4	Traditional	No
Pu8	9	Female	Community open area	5	3	Traditional	No
Pu9	10	Male	Home	1–3	4	Traditional	Yes
Pu10	11	Female	Community open area	1–3	4	Traditional	No
Pu11	10	Female	Home	3–5	4	Countable	Yes
Pu12	11	Female	Community open area	7	5	Traditional	Yes
Pu13	10	Male	Community open area	5	4	Traditional	No

## Appendix B

**Table A2:** Parents characteristics.

Participant Number	Age	Gender	Occupation	Child’s Age	Frequency of Parent-Child Paired Physical Activity (Times/Week)	Teaching Experience
Pa1	45	Female	Project Manager	9	1–2	Yes
Pa2	52	Male	Engineer	11	2	Yes
Pa3	46	Female	Freelancer	8	3–5	Yes
Pa4	39	Male	Programmer	7	1–3	Yes
Pa5	40	Female	Freelancer	9	1–2	Yes
Pa6	37	Female	Housewife	7	5	Yes

## Appendix C

**Table A3:** Procedure of user research.

Method	Participant
Observation	Pu1 & Pa1
Observation & Semi-structured interview	Pu2 & Pa2
	Pu3 & Pa3
	Pu4 & Pa4
	Pu5 + Pa5
Semi-structured interview	Pu6 + Pa6
	Pu7
	Pu8
	Pu9
	Pu10
	Pu11
	Pu12
	Pu13

## Appendix D

**Table A4:** Usability test tasks.

Task	Material	Word of Task Guiding
Task 1: Initial impression of the application and to view the TAB of the menu bar		What are the functions of the four tabs in the home menu bar? Is it appropriate?
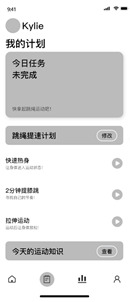
Task 2: To make a new exercise plan		How to make a new plan? Whether the information required is understood?
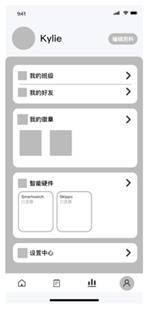
Task 3: To read exercise guidance		Which button to press to read exercise guidance? Is it easy to understand? Is the interface text size of the guidance page appropriate?
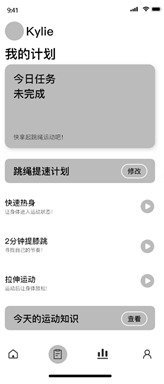
Task 4: To start exercising on smart watch		How to start exercising? Is the introduction text size appropriate? Is the guidance animation effective?
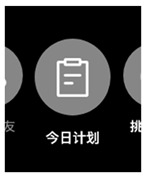
Task 5: To examine exercise data		What do the icons on this calendar mean? How to examine today’s exercise data? What data are recorded? Are they necessary? How to switch from daily data analysis to weekly data analysis?
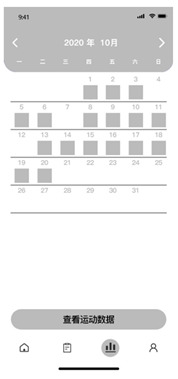
Task 6: My Garden interface		What is the function of this interface? How to water my plant? How to examine my harvest? How do I know the growing progress of my plants?
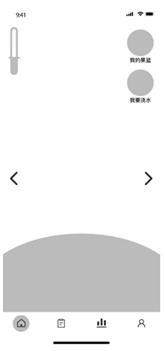
Task 7: To interact with friends		How to add friends? How to check your friend’s exercise status on two devices? Is ranking necessary? How to give a “thumb up“ to your friend?
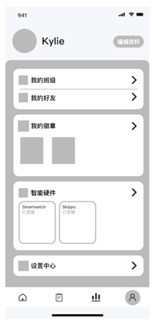
Task 8: Personal homepage function interface		What is the function of the personal homepage interface? How to examine the connection status of hardware and software devices? How to add my class?

## References

[1] WHO (2010). Global Recommendations on Physical Activity for Health.

[2] Guthold R., Stevens G.A., Riley L.M., Bull F.C. (2020). Global Trends in Insufficient Physical Activity among Adolescents: A Pooled Analysis of 298 Population-Based Surveys with 1·6 Million Participants. Lancet Child Adolesc. Health.

[3] Yang X., Lee J., Gu X., Zhang X., Zhang T. (2020). Physical Fitness Promotion among Adolescents: Effects of a Jump Rope-Based Physical Activity Afterschool Program. Children.

[4] Trecroci A., Cavaggioni L., Caccia R., Alberti G. (2015). Jump Rope Training: Balance and Motor Coordination in Preadolescent Soccer Players. J. Sport Sci. Med..

[5] Ha A.S., Lonsdale C., Ng J.Y.Y., Lubans D.R. (2017). A school-based rope skipping program for adolescents: Results of a randomized trial. Prev. Med..

[6] Chon T.J., Sung D.J., Jeon J.Y., Shin J.T. (2018). Enhancing Psychological and Physical Fitness Factors of Korea Middle School Students by Introducing Rope Skipping. Iran. J. Public Health.

[7] Wang Y., Shen Y. (2015). Investigation on the present situation of primary school skipping sport development in Kunming city. J. Kunming Univ..

[8] Jump Rope for Heart Initiative. http://www.sport.gov.cn/n316/n343/n1191/c581373/content.html.

[9] Staiano A.E., Calvert S.L. (2011). Exergames for Physical Education Courses: Physical, Social, and Cognitive Benefits: Exergames for Physical Education Courses. Child Dev. Perspect..

[10] Boon B., Rozendaal M., van den Heuvel-Eibrink M., van der Net J., van Grotel M., Stappers P. (2020). Design Strategies for Promoting Young Children’s Physical Activity: A Playscapes Perspective. Int. J. Des..

[11] Deterding S., Dixon D., Khaled R., Nacke L. (2011). From game design elements to gamefulness. Proceedings of the 15th International Academic MindTrek Conference: Envisioning Future Media Environments.

[12] Williams W.M., Ayres C.G. (2020). Can Active Video Games Improve Physical Activity in Adolescents? A Review of RCT. Int. J. Environ. Res. Public Health.

[13] Maric D., Kvesic I., Lujan I.K., Bianco A., Zenic N., Separovic V., Terzic A., Versic S., Sekulic D. (2020). Parental and Familial Factors Influencing Physical Activity Levels in Early Adolescence: A Prospective Study. Healthcare.

[14] Gao Z., Chen S. (2014). Are field-based exergames useful in preventing childhood obesity? A systematic review. Obes. Rev..

[15] Lwin M.O., Malik S. (2012). The efficacy of exergames-incorporated physical education lessons in influencing drivers of physical activity: A comparison of children and pre-adolescents. Psychol. Sport Exerc..

[16] Gao Z., Podlog L. (2012). Urban Latino children’s physical activity levels and performance in interactive video dance games: Effects of goal difficulty and goal specificity. Arch. Pediatr. Adolesc. Med..

[17] Garnweidner-Holme L.M., Borgen I., Garitano I., Noll J., Lukasse M. (2015). Designing and Developing a Mobile Smartphone Application for Women with Gestational Diabetes Mellitus Followed-Up at Diabetes Outpatient Clinics in Norway. Healthcare.

[18] Chan M., Esteve D., Fourniols J.Y., Escriba C., Campo E. (2012). Smart wearable systems: Current status and future challenges. Artif. Intell. Med..

[19] Mayordomo-Martínez D., Sánchez-Aarnoutse J.C., Carrillo-de-Gea J.M., García-Berná J.A., Fernández-Alemán J.L., García-Mateos G. (2019). Design and Development of a Mobile App for Accessible Beach Tourism Information for People with Disabilities. Int. J. Environ. Res. Public Health.

[20] Yu N., Huang Y.T. (2020). Important Factors Affecting User Experience Design and Satisfaction of a Mobile Health App—A Case Study of Daily Yoga App. Int. J. Environ. Res. Public Health.

[21] Grand View Research (2018). mHealth Market Size, Share, Global Industry Trends Report, 2018–2025. https://www.grandviewresearch.com/industryanalysis/mhealth-market.

[22] Tate E.B., Spruijt-Metz D., O’Reilly G., Jordan-Marsh M., Gotsis M., Pentz M.A., Dunton G.F. (2013). mHealth approaches to child obesity prevention: Successes, unique challenges, and next directions. Behav. Med. Pract. Policy Res..

[23] Sanders J.P., Loveday A., Pearson N., Edwardson C., Yates T., Biddle S.J., Esliger D.W., Lyden K., Miner A. (2016). Devices for Self-Monitoring Sedentary Time or Physical Activity: A Scoping Review. J. Med. Internet Res..

[24] McCallum C., Rooksby J., Gray C.M. (2018). Evaluating the Impact of Physical Activity Apps and Wearables: Interdisciplinary Review. JMIR mHealth uHealth.

[25] Källander K., Tibenderana J.K., Akpogheneta O.J., Strachan D.L., Hill Z., ten Asbroek A.H., Conteh L., Kirkwood B.R., Meek S.R. (2013). Mobile health (mHealth) approaches and lessons for increased performance and retention of community health workers in low- and middle-income countries: A review. J. Med. Internet Res..

[26] Corpman D.W. (2013). Mobile Health in China: A Review of Research and Programs in Medical Care, Health Education, and Public Health. J. Health Commun. Int. Perspect..

[27] Ni Z., Liu C., Wu B., Yang Q., Douglas C., Shaw R.J. (2018). An mHealth intervention to improve medication adherence among patients with coronary heart disease in China: Development of an intervention. Int. J. Nurs. Sci..

[28] Big Data Research (2019). 2019Q1 Mobile Medical Report.

[29] Kairy D., Mostafavi M.A., Blanchette-Dallaire C., Belanger E., Corbeil A., Kandiah M., Wu T.Q., Mazer B. (2021). A Mobile App to Optimize Social Participation for Individuals with Physical Disabilities: Content Validation and Usability Testing. Int. J. Environ. Res. Public Health.

[30] Davidson C. (2009). Transcription: Imperatives for qualitative research. Int. J. Qual. Methods.

[31] Hunt M.R. (2009). Strengths and challenges in the use of interpretive description: Reflections arising from a study of the moral experience of health professionals in humanitarian work. Qual. Health Res..

[32] Giacomin J. (2014). What Is Human Centred Design?. Des. J..

[33] Pruitt J., Adlin T. (2006). The Persona Lifecycle: Keeping People in Mind throughout Product Design.

[34] So C., Joo J. (2017). Does a Persona Improve Creativity?. Des. J..

[35] Eather N., Morgan P.J., Lubans D.R. (2013). Social support from teachers mediates physical activity behavior change in children participating in the Fit-4-Fun intervention. Int. J. Behav. Nutr. Phys. Act..

[36] O’Reilly E., Tompkins J., Gallant M. (2001). ‘They Ought to Enjoy Physical Activity, You Know?’: Struggling with Fun in Physical Education. Sport Educ. Soc..

[37] The Color Psychology of Orange. https://www.verywellmind.com/the-color-psychology-of-orange-2795818.

[38] Planning a Usability Test. https://www.usability.gov/how-to-and-tools/methods/planning-usability-testing.html.

